# Adaptations to Holiday Club Food Provision to Alleviate Food Insecurity During the Covid-19 Pandemic

**DOI:** 10.3389/fpubh.2021.661345

**Published:** 2021-09-30

**Authors:** Natasha Bayes, Clare E. Holley, Emma Haycraft, Carolynne Mason

**Affiliations:** School of Sport, Exercise and Health Sciences, Loughborough University, Loughborough, United Kingdom

**Keywords:** holiday clubs, staff insights, food insecurity, holiday hunger, Covid-19

## Abstract

Holiday clubs play a pivotal role in providing food and vital enrichment opportunities to alleviate food insecurity among children during the school holidays (holiday hunger). The need for these opportunities increased substantially for families throughout 2020, as food insecurity quadrupled in the UK during the Covid-19 pandemic. In this qualitative study, holiday club staff from England and Wales reflected on the adaptations they implemented in order to maintain food supplies and food-related enrichment activities for families during the first UK national Covid-19 lockdown and subsequently throughout the summer of 2020. Staff also reflected on the opportunities and challenges related to implementing these adaptations during this period. Twenty-five holiday club staff engaged in video-based interviews during August and September 2020. The findings revealed a range of innovative changes to holiday club food provision, and the challenges and opportunities faced varied across holiday clubs. Challenges during the pandemic in some clubs included staff shortages (typically due to furloughing and/or increased working demands) and difficulties sourcing adequate funding. However, staff identified that the opportunities for holiday clubs included enhanced partnership working during the pandemic, increased engagement with digital technology to communicate with families and deliver their online cooking sessions, and their ability to continue providing food and much needed creative opportunities for children unable to attend school and/or the holiday club. The ability of clubs to adapt their models of working when faced with adversity was essential in protecting their organisational resilience and delivering their vital services. The findings emphasise the important role that holiday clubs play in their communities and highlight their willingness to adapt and expand their role in response to the pandemic to continue to tackle food insecurity and provide vital food and food-related enrichment opportunities to families. The findings also identify lessons that can be applied to practise in the future.

## Introduction

In March 2020, the World Health Organisation (1) declared the novel coronavirus [SARS-CoV-2 virus, Covid-19 disease; (2)] outbreak to be a pandemic, and this remains of global concern. In the UK, the first national lockdown began on 26th March 2020 to manage the spread of the virus (3), resulting in schools closing to all children except vulnerable children and those of key workers (4). Before the pandemic, ~10% of families were experiencing severe food insecurity (5, 6), and food insecurity was consistently on the rise prior to Covid-19 (5, 7, 8). After the onset of the pandemic, food insecurity was 250% higher by May 2020 compared to pre-Covid-19 (9), with single mothers and low-income households being most financially impacted during this period (10, 11). Alongside this, demand for emergency food aid increased significantly. The Trussell Trust, a UK national foodbank network, estimated a 107% increase in the distribution of food parcels to children in April 2020 compared to the year before (8, 12). Additionally, between April and June 2020, almost 100,000 new households received food aid *via* the Trussell Trust for the first time (8). Families experiencing food insecurity during the pandemic were at risk of inadequate dietary quality and quantity, compromised food safety, changes in weight status, increased anxiety and depression, and a range of other food insecurity fuelled impacts during the Covid-19 pandemic (7).

The causes of increased food insecurity during the first UK national Covid-19 lockdown were multi-layered and complex. Affordable food became less accessible (13), as food increased in price, stocks were in shorter supply due to panic and bulk buying, and there was reduced access to local discount shops selling food items cheaply due to lockdown-enforced shop closures (7). Basic living costs rose due to rising food costs and rising utility bills from caring for families and/or working from home (7). Household income decreased for many due to unemployment, reduced working hours and redundancies driven by the national economic fallout of Covid-19 (14). The Trussell Trust (8) reported that low-income remained the predominant reason for foodbank referrals in 2020, following the onset of the pandemic. Alongside this, the government-enforced closure of schools increased the risk of free school meal (FSM) eligible children experiencing greater food insecurity without access to a meal within the school day. In response to the increased risks of children's food insecurity, on 31st March 2020, free school meals were replaced with food parcels and an adjacent voucher scheme enabling families to shop in specific supermarkets (4). However, these schemes were problematic and only marginally protected families from food insecurity; the £15 per week per FSM eligible child provided insufficient funds for families to feed their children nutritious meals, access to FSM vouchers was frequently delayed due to technological issues inducing delays in claims processing, and vouchers were initially only eligible for use in specific supermarkets, some of which were inaccessible to some families (4). Research also found that 49% of FSM eligible children did not receive the FSM voucher scheme, concluding that the voucher scheme was not a sufficient replacement for FSM provision (15).

Alongside the FSM vouchers, various local and national government and charity organisations provided food to families to alleviate food insecurity exacerbated by the Covid-19 pandemic. The Department for Education's (DfE) Holiday Activity and Food (HAF) programme and other charity and local government organisations funded holiday programmes/clubs to alleviate food insecurity and provide important food (e.g., cookery) and non-food (e.g., sport, physical activity) related enrichment opportunities during the school holidays (16, 17). The DfE's HAF programme was first implemented in 2018, providing £2 million to pilot the programme (18, 19), although some holiday clubs have operated in disadvantaged communities prior to the inception of the HAF funding. In 2020, its funding reached ~50,000 FSM eligible children across 10 geographic areas, falling short of the 1.3 million children eligible for FSMs (4, 20). Non-DfE funded holiday programmes are therefore also essential to protect families outside of the DfE targeted areas and yet it is currently unknown how many non-DfE funded programmes are operating. Nevertheless, prior to Covid-19 research suggested that holiday clubs have a range of benefits including alleviating hunger (17), reducing social isolation (21), enhancing social skills (22), and promoting healthy eating (23) and physical activity (21). Similarly, holiday clubs provide a wealth of services including material goods (e.g., food and entertainment equipment), sport, physical activity, fun days out, information and education (24). Holiday programmes are therefore pivotal to the health and wellbeing of some of the most vulnerable children, and undoubtedly this has remained the case during the Covid-19 pandemic given the significant and accelerated rise in food insecurity. Yet little is currently known about how holiday clubs in England/Wales have sustained their food supplies to families in disadvantaged communities during the pandemic, and how they have responded to the changes in demand for food and the enforcement of government rules and regulations to manage the spread of Covid-19, such as the national lockdown restrictions, localised rules and restrictions, and the need to universally practise social distancing (25, 26). The aim of this study was firstly to explore the adaptations made by holiday clubs in order to maintain their food supplies and food-related enrichment activities for families during the first UK national Covid-19 lockdown and subsequently throughout the summer of 2020. Secondly, this study examined the opportunities and challenges holiday clubs faced in making these adaptations. Third and finally, this study captured the learning from these experiences to further empower holiday clubs to achieve their aims of supporting families from disadvantaged communities. In this study, the term “holiday clubs” refers to play schemes ran during the school holidays by various private, government, voluntary or community organisations, which offer food and food-related enrichment opportunities to children from food insecure backgrounds.

## Materials and Methods

### Research Philosophy and Ethics

This study was designed and implemented within an interpretivist research paradigm using qualitative methods (semi-structured interviews). The data obtained for this study was collected as part of a larger study which aimed to explore the feasibility of holiday club staff implementing evidence-based feeding practises to promote healthy eating in children attending holiday clubs. As the feasibility study was conducted during the Covid-19 period, conversations about Covid-19 were timely and emerged organically within the video-based interview process. During these conversations, staff reflected on the adaptations they implemented in order to maintain food supplies and food-related enrichment activities to families during the first UK national Covid-19 pandemic period (March–May 2020) and subsequently throughout the summer of 2020, and the opportunities and challenges related to implementing these adaptations.

This study received ethical approval from the Loughborough University Ethics Committee (ref: SSEHS-1644).

### Participants

Participants were staff implementing England/Wales holiday club programmes during the school holiday periods. During the early stages of the Covid-19 pandemic (from March 2020, with UK national lockdown measures between March and May 2020), many clubs extended their provision during both school term and holiday periods due to the lockdown-enforced school closures.

Staff were over the age of 18 and conducting either a paid or voluntary role within the club. Holiday clubs typically involved primary school aged children (e.g., age 5–11 years old), with broader age groups in some clubs (e.g., 0–16 years). Staff were working in clubs with a high proportion of free school meal (FSM) eligible children attending the club, of which FSM eligibility is an indicator of deprivation and increased risk of food poverty.

Twenty-five staff engaged in this study from 24 different clubs located in nine regions within England/Wales (Table 1), and ran in community settings including community centres, sport centres, schools, and churches. All of the staff engaged were from clubs that continued to provide food to children during the first UK national lockdown and through the 2020 summer holidays.

**Table 1:** Club staff regional localities.

**Region**	***n***
East Midlands	7
Yorkshire and the Humber	4
South East	4
South West	3
North West	2
West Midlands	2
London	2
East of England	1
Wales	1

### Recruitment and Data Collection

The researcher (NB) conducted desk-based research to develop a contact database of local and national charity and government organisations (*N* = 40) overseeing holiday club programmes offering food as part of their programme provision for children living in disadvantaged communities. It is not known exactly how many holiday clubs/providers exist across England and Wales as no single list of the clubs exists. Estimates in 2017 suggest there are 593 clubs, and half of these were run by voluntary organisations (27). It is known that these have become more commonplace and the number is rising in response to heightened food insecurity.

The researcher telephoned and emailed organisations on the contact database requesting their recruitment support. A total of 15 local (*n* = 9) and national (*n* = 6) charity/non-profit community interest company organisations agreed to facilitate participant recruitment (see acknowledgments), by circulating a study poster to clubs implementing their club programme provision *via* email and/or social media (Twitter and Facebook). Club staff who were interested in participating in the study were asked to complete an online survey consisting of a full participant information sheet, consent form, name and contact information questions, and demographics questions asking their age, gender, ethnicity, nutritional qualifications, club name and postcode, role and length of time employed with the club, and the average number and average age range of child attendees within the club.

Participants who had expressed their willingness to join the study through completing the online survey were contacted by the researcher *via* telephone and email to organise a date and time to engage in an online video interview. Telephone interviews were conducted with a small number of participants without the relevant technology and/or when there were issues with internet connectivity.

A pragmatic approach was adopted to determine an acceptable sample size for this study, a method recognised in current qualitative methodology literature (28).

Interviews lasted between 26 and 71 min. Interviews were recorded and facilitated by the lead researcher. As the data for this study was collected as part of a feasibility study, a formal semi-structured interview schedule was utilised during interviews for the feasibility study. There was no formal interview schedule for this present study as the discussions related to Covid-19 were driven by the participants and, given the significance of Covid-19 during the study period, the researcher allowed these discussions to emerge naturally and asked further questions for elaboration and clarification when they emerged. However, conversations were typically based around adaptations made to holiday club food and food-related enrichment provision during the early months of the Covid-19 pandemic, and the opportunities and challenges experienced in implementing these adaptations.

### Data Analysis

Interviews were transcribed verbatim and analysed, guided by Braun and Clarke's (29) 6-step thematic analysis framework. Thematic analysis complements research positioned within an interpretivist paradigm (29, 30), and provides flexibility through the analysis process while also having the potential to generate a rich and detailed account of data (29). The thematic analysis was conducted using the analysis software NVivo, enabling the data to be organised into generated codes and themes.

The thematic analysis was conducted using a bottom-up, data driven inductive approach (31), as there is no current literature available to draw upon on the adaptations made and the opportunities and challenges of these adaptations in holiday clubs during the first Covid-19 lockdown and 2020 summer holidays. The analysis process utilised the combined academic experiences of the research team who have conducted previous research with holiday clubs over an extended period prior to the Covid-19 pandemic. The initial thematic framework that was developed included the themes of Adaptations, Opportunities, Challenges, and Implications but it became apparent that these themes were strongly interlinked and that experiences varied significantly from one club to another. The thematic framework therefore evolved to focus more specifically on providing a detailed account of the “Adaptations” theme paying particular attention to identifying the changes that were made and the reasons for the change. This then enabled the opportunities, challenges and implications of these adaptations to be identified.

The lead researcher read each transcript to become familiar with the data. Transcripts were then coded, and these codes were organised into an initial thematic framework. Transcripts were further coded and recoded as the coding and thematic framework evolved. Additionally, research team members (CM, CEH, and EH) reviewed and provided feedback on the thematic framework to improve its structure and robustness.

## Results

### Descriptive Information About the Holiday Club Staff

The club staff (*N* = 25) who participated in the study ranged from 23 to 69 years old (mean = 48.08 years) and were predominantly female (*n* = 18, 72%) and White British (*n* = 23, 92%). A small proportion of staff had a nutrition qualification (*n* = 8, 32%), including food hygiene, nutrition for sport and physical activity, and nutrition for the catering industry. The number of years working at the club varied from 1 to 25 years (mean = 4.68 years). The staff roles were varied and often involved conducting dual roles, including chief executive, club manager, project lead, coordinator, administrator, youth worker, and cooking and catering staff.

### Thematic Analysis

Holiday club staff insights about their response to Covid-19 were explored in relation to the adaptations made in order to maintain food supplies to families, and the opportunities and challenges related to implementing these adaptations. The analysis revealed three key themes: (1) Changes to food delivery methods; (2) Changes to the reach and scale of food provision, and; (3) Changes in resources. These themes are described below.

#### Changes to Food Delivery Methods

##### Reasons for Change

Government rules and guidelines implemented to manage the spread of Covid-19 (e.g., the national lockdown restrictions, localised rules and restrictions, the need to universally practise social distancing) required the clubs to adapt their current models of working to ensure compliance with these enforced restrictions. For some clubs, adaptations enabled them to continue their on-site food provision for children and families, while other clubs postponed their on-site provision and created new ways of offering food to children and families off-site.

Staff highlighted that “*we couldn't do it the same way it has been done in the previous years, but it was still feeding children*” (P14, Planner). However, some staff highlighted that regular revisions to local and national government rules and restrictions created uncertainty about how best to deliver the club provision and required club staff to frequently reassess and adjust their models of working in line with current government guidelines:

“*So at the moment we're not allowed to meet. But we're hoping in a couple of weeks, restrictions might have lifted a little bit, and once schools are back into like, some kind of routine, then maybe we can start some kind of food project again. So yeah, we will definitely be doing, well I say definitely, but nobody knows for definite. But even during the actual lockdowns, we couldn't run the actual holiday club, but we still met and did the cooking, but it then got doorstep delivered to the families. That's what we did in the summer. We opened for a week, and then we got closed because the restrictions got raised in [local area name removed]*” (P4, Chief Executive).

##### On-Site Provision

Of the clubs who continued to offer on-site club provision, the staff highlighted the importance of adapting their food provision to provide for children and families in Covid-compliant ways. Some staff highlighted that while they had previously offered hot meals in the club, they had to adapt their meal provision to lunch bags with cold food items “*like a roll, a sandwich and fruit and a snack*” (P14, Planner). One member of staff highlighted that these changes were necessary as their hot buffet meal provision would create “*cross contamination issues*” (P4, Chief Executive) due to the Covid-19 rules to maintain social distancing and reduce high-touch areas, which they would find difficult to monitor and manage.

Staff highlighted numerous benefits to these adaptations from hot meals to cold food lunch bags, including making it “*much more easy to be flexible around what goes in them [the lunch bags]*” (P2, Coordinator) and simplifying the meal serving process, such as reducing the amount of unpaid volunteers to coordinate each day, and reducing food hygiene risks associated with preparing and serving hot food on site. Although cold food provision was logistically simpler, Covid-compliant, and enabled clubs to continue to feed children, some staff were concerned that this change did not afford children the benefits that a hot meal offers:

“*The thing I have a real concern about is that it's great that we can do them packed lunches because it means we can deliver the service, but I do worry about children not having hot meals. I think that the need to sit down and have a hot meal is super important. Also, it does force you to sit down to eat, which cold food doesn't*” (P2, Coordinator).

##### Off-Site Provision

Where clubs found it too difficult to continue to provide food on-site, staff identified alternative ways to provide food to children and families outside of the club setting. Delivering food and recipe boxes was a particularly popular method adopted by most clubs to enable them to continue to provide food to families:

“*We are sending out a weekly pack of food and craft activities. And with the food they will get recipes and ideas to do with the food that we are providing*” (P5, Coordinator).

In providing food and recipe boxes, staff carefully considered what items to include in the boxes. Staff reflected on the importance of providing healthy, non-perishable, staple foods that were reasonably priced to ensure the recipes could be replicated in the future by families. One member of staff also highlighted the importance of ensuring the boxes were tailored to meet the dietary needs of each individual family. Additionally, one staff member discussed the challenges and solutions made for families who did not have access to the kitchen utensils required to prepare and cook the meals:

“*The things that we've discovered is a lot of our families don't have kitchen scales. And our school meals service have made up a utensils' library, and we've got some scales in there. But for the summer, where we didn't have access to that, we measured things out because we knew how many were in each family*” (P11, Coordinator).

Staff experienced challenges related to creating and delivering the food and recipe boxes. Staff had to ensure this was completed in Covid-compliant ways such as “*gelling hands and wearing face masks*” (P6, Leader/Coordinator) when delivering the boxes to families, which made these activities more time consuming. One member of staff also highlighted that “*of course, by the nature of the families, there's always the odd one who wasn't home when they'd promised to be, so that took a bit of extra time*” (P6, Leader/Coordinator). Despite the challenges, staff felt that the boxes were important for providing families with recipe ideas that could be replicated in the future and for “*offering an opportunity for parents to try and make that recipe at home with their kids*” (P14, Planner).

Similar to the food and recipe boxes, one club batch cooked and froze their hot meals and delivered these to families, enabling them to have a ready-made hot meal. A few clubs also described a seasonally themed food offering for families:

“*During the school holiday, we've been providing picnics. So we sort of made sandwiches, fruit and drink, and then they [parents] did actually book those and came and collected a picnic for however many were in their family. And we're also able to, because we've had some funds, we gave them some toys, some things like balls and frisbees so that they had something to play with. That went down really well as well*” (P9, Planner/Caterer).

Staff highlighted that “*families have missed the social interaction*” (P11, Coordinator) that is afforded through on-site club provision. Although holiday clubs preferred to deliver their provision on-site, in light of the current crisis, they felt happy to be able to provide food supplies to families through new delivery methods. Importantly, staff reflected on the importance of maintaining food resources to struggling families:

“*The families were very appreciative, and they expressed their appreciation and, you know, the conversations we've had they said, if we hadn't provided them that, they would have really struggled over the summer. So it was rewarding to think that it was necessary and that they appreciated it*” (P6, Leader/Coordinator).

##### Interactive Food Activities

Pre-Covid, many clubs regularly engaged children and/or families in food preparation and cooking enrichment activities within the holiday club setting. The pandemic created new challenges in involving families in these interactive activities as it was difficult to adapt them to be delivered in Covid-compliant ways. Maintaining government rules related to social distancing was considered particularly challenging when involving children and families in cooking activities within the club setting and this resulted in cooking activities being postponed in many clubs. However, some clubs were able to adapt methods of involving families in cooking activities in Covid-compliant ways, enabling these activities to be delivered on-site. These adaptations included involving smaller numbers of families in these activities at one time and providing each family with their own cooking utensils to be used exclusively by them:

“*With the cooking, they can make their own biscuits in their own bowls that they keep for themselves and they can't share, which would be different from how we would normally do it*” (P4, Chief Executive).

Many of the staff highlighted that as on-site cooking activities were not possible, they adapted their practises to enable families to engage in cooking activities through the delivery of the food and recipe boxes combined with the delivery of virtual cooking sessions, either through live streaming the session or through pre-recording cooking demonstration videos and hosting them online for families to access. One member of staff reflected on the benefits of offering these virtual cooking sessions, commenting that they “*bring something fresh and new and a focus each week*” (P18, Coordinator) for families trapped in the mundane routine of lockdown, and also that they were associated with changes to children's eating behaviours:

“*And another mum said her child, because he'd been involved in the cooking, would then eat it, whereas he [usually] was really fussy. So you know, really small changes that actually long term, will have quite a big impact*” (P18, Coordinator).

##### Virtual Communication and Engagement

Most of the staff discussed the important role that digital technology played in facilitating the delivery of their club provision. Staff communicated with parents through a range of digital mediums, including text messaging and social media platforms such as Facebook, about various things related to the club provision:

“*We asked permission and got their consent to get some details about allergies and food preferences or dietary requirements, or health related problems that would affect their diet. So, we were able to shop appropriately for each family and individualise it*” (P6, Leader/Coordinator).

Many staff highlighted that Facebook was a valuable platform to communicate effectively with parents and to host/link cooking demonstration videos for families to access to facilitate their engagement in cooking activities:

“*And we set up our own Facebook page which we then linked to a YouTube page, and then I demonstrated on video how to make the dishes that were in the recipe bags. So we had chicken Fajitas, beef and bean bake, we had sausage risotto, we had frittata, and we had muffin pizzas. So we did a video and we put those up online*” (P12, Project Lead/Cook).

Facebook also provided a platform for families to share photos and videos of their child(ren) involved in cooking activities at home, which helped to foster a sense of community in the absence of in-person interactions.

The staff highlighted opportunities and challenges with using technology to communicate and engage with families. Offering cooking demonstration videos online provided enrichment and engagement opportunities to families, and a platform where “*the families are learning together*” (P5, Coordinator). Access to these online videos was pivotal for enabling clubs to continue to reach and engage families in food activities when on-site club provision was postponed or when families “*can't leave the house or go to any of the activities*” (P14, Planner), for example if they were shielding or self-isolating.

Although the use of digital platforms was considered essential to engage with families during the Covid-19 pandemic, some staff felt engaging children using digital platforms was generally more appropriate for older children (e.g., 12+) than younger children, and that it was more difficult engaging children when they are at home rather than face to face:

“*Well, we know how it's been working from home that, when you've got, you know, other people in the house, and other things going on, how easily it is to get distracted*” (P4, Chief Executive).

Some staff also highlighted challenges for some families in accessing digital technology:

“*And we talk about ‘oh well, you can go online,' but again it's a choice between credit on a phone or food in their bellies. So we really have to think about when we are saying things like that and what impact it might have and what additional pressures we might put on those families*” (P3, Manager).

However, one member of staff highlighted that in order to promote engagement and inclusivity to all families, including those who are hardest to reach and with limited access to digital technology, they had created a “*play book and food book which gives them ideas of different foods the young people can make at home. It also has the nutritional value, how to make the food and the benefits of that food*” (P7, Programme Manager).

#### Changes to the Reach and Scale of Food Provision

##### Reasons for Change

Many staff reflected on the changes to families' personal circumstances that occurred during the Covid-19 pandemic. Changes in working and financial circumstances were particularly prevalent. Many families experienced redundancy, furloughing or requirements to work from home. One staff member highlighted that some families became more financially secure during the pandemic as the furlough scheme afforded them with consistent financial resources. However, most staff reported that the changes in employment circumstances typically resulted in increases in financial insecurity and consequently increases in food insecurity among families, for a multitude of complex reasons such as low income, lack of eligibility for free school meals, slow processing of new state benefit claims, and slow implementation of the national School Meals Voucher scheme during 2020. For example:

“*We started to pick up families who had been made redundant and universal credit wasn't kicking in and they weren't on the list therefore [not eligible] for free school meals, and so missed the summer school meal voucher programme and were in quite a desperate position really*” (P12, Project Lead/Cook).

Such changes in personal circumstances resulted in new and existing families seeking support from the holiday clubs for food supplies. One staff member “*was quite concerned for the number of families who were finding themselves in desperate circumstances for the first time*” (P12, Project Lead/Cook) as these families often had to navigate unfamiliar crisis services such as foodbanks and the state benefits process. Another staff member reflected concern on the impact that these increases in food insecurity would have on services:

“*We are now, unfortunately, looking at the next wave of redundancies and things of that nature that will impact massively on the services, and it will stretch us as an organisation and other organisations across the country delivering those sorts of things*” (P3, Manager).

##### Scale and Reach of Provision

While many staff reflected on the increase in families' need for support from the holiday club, the staff described a variety of changes in the scale and reach of their food provision during the Covid-19 pandemic period. In some clubs, their provision decreased. One staff member highlighted concerns that, due to postponing their on-site provision, they would not be able to reach and engage as many children and families despite the adoption of digital methods to engage families. Another staff member highlighted that after the first lockdown, they were able to resume their on-site club provision, but noticed a reduction in attendance rates due to increased fears of the spread of Covid-19:

“*Kids maybe didn't come straight away because parents were worried about kids doing something because, you know, they've been in lockdown for so long*” (P14, Planner).

In many of the clubs, the number of meals provided, and number of families supported, increased significantly during the pandemic:

“*So at the moment we deliver, we do about 218, well we say meals, but it means, it's a bag of food which would make that many meals. So during a normal week, we'd normally do about 218 meals, but during Covid, we were doing about 600 a week*” (P4, Chief Executive).

Some staff highlighted that the adaptations made to their service delivery methods from on-site to off-site meal provision enabled them to increase the volume of food provision to reach more families:

“*Obviously because of Covid we had to adapt our delivery and that was a major change, and a really positive thing that came out of it was that we could offer our services to the community to deliver food packages county-wide, so we weren't restricted to any areas and the funding didn't provide any barriers in that respect. It just opened up our reach to the community really, so that was really positive*” (P8, Senior Youth Worker).

While these increases in food provision highlight that clubs adapted to stretch their services to families requiring support, the need to increase their provision is symptomatic of the increase in families experiencing food insecurity and requiring emergency food provision. As reflected by some staff, families seeking support were “*everything from our longest standing family who have been with us from day one, right through to strangers we didn't know just calling our helpline and asking for help*” (P12, Project Lead/Cook).

#### Changes in Resources

The staff described changes to numerous club resources which impacted their food provision during 2020, including changes in staffing, facilities, and finances as well as changes in partnership working within the club and among external stakeholders.

Most staff reflected on how partnership working was strengthened during the Covid-19 pandemic period. Increased partnership working enabled some clubs to access additional funding resources, and enabled knowledge exchanges about different recipe ideas and different ways of delivering their club provision. Additionally, increased partnership working with food partners such as schools, local supermarkets and food surplus organisations such as FareShare provided clubs with additional food donations, enabling more families to access food:

“*There was FareShare food that was available free of charge, so that meant that we could carry on and provide for the extra families, which proved quite useful*” (P6, Leader/Coordinator).

Staff sometimes highlighted that the food donations received were sometimes heavily processed ambient goods and lacking in fresh and healthy foods. Some staff also highlighted that food donations were sometimes towards the end of their shelf life, which meant some foods were wasted, and sometimes donations were large volumes of snack foods (typically high calorie, low nutritional value) which meant they were slow to redistribute due to a desire to minimise the volume of snack foods provided to families:

“*Oh and we were given hundreds and hundreds of packets of biscuits. I had 941 packets of digestives that's lasted us since April, which we've now just got rid of [August]. And then we had about 400 packets of chocolate digestives and fruit shortcakes*” (P12, Project Lead/Cook).

Staff reflected on diminished resources including reductions in staffing due to furloughing, increased working demands among some staff, decreased access to club kitchen facilities and difficulties raising funds to financially underpin the club provision. Some staff highlighted that despite these resource challenges, they were “*coping*” (P5, Coordinator) with the demands of the club provision, while others highlighted that they found it difficult to provide the fresh and healthy produce that families needed:

“*If I could afford to put more fresh fruit and veg in our boxes that we send out, if we could give our families a bag of fresh fruit and veg to take home. I asked them ‘if you could have one thing in your boxes, and I'm not guaranteeing you're going to get it, what would it be?' And I would say that about 90% of the families said fresh fruit. It was great that we could put out tinned fruit, and send them some packet custard, because at least it's getting some nutrient into them, but, yeah, to have that sort of thing [fresh fruit and vegetables] would be really good*” (P12, Project Lead/Cook).

However, many staff highlighted their access to resources was sufficient. Some staff already had the necessary volume of staff in place to oversee the running of the food provision and effectively respond to the Covid-19 crisis, while others highlighted an increase in volunteers, sometimes as a result of people being furloughed from their usual jobs which gave them more time to help with the delivery of the club provision. Similarly, many staff accessed the necessary financial resources needed to obtain and sustain sufficient food supplies and support additional families with their food provision. These financial resources were underpinned by the efforts of staff in sourcing additional funding and by increasing their resourcefulness such as “*bulk buy[ing] and split[ting]*” (P11, Coordinator) which was more time consuming for staff but enabled cost-effective food provision. One staff member highlighted that while they received sufficient financial resources to alleviate food poverty during the start of the crisis, they do not typically receive these resources and were concerned that this financial support would unlikely be available in the future:

“*During Covid there has been funding made available to relieve food poverty, which is the first time in 4 years that we've ever seen money for this. But that will stop, and I assume, you know, there's not an endless pot of funding. So we have been able to get some funding to do this during this period, which has helped. But long term, yeah, how do you generate a revenue around this, it's really, really tricky*” (P4, Chief Executive).

## Discussion

This study sought to explore the adaptations made by England/Wales holiday clubs in order to maintain food supplies and food-related enrichment activities for families during the first UK national Covid-19 pandemic period (March–May 2020) and subsequently throughout the summer of 2020. The study also explored the opportunities and challenges faced in making these adaptations and captures the learning from these experiences to further empower holiday clubs to achieve their aims of supporting families from disadvantaged communities. To our knowledge, this is the first study to explore how holiday clubs have responded to the challenges faced in providing food to families during the Covid-19 pandemic. The findings revealed three key themes: (1) Changes to food delivery methods; (2) Changes to the reach and scale of food provision; and, (3) Changes in resources. These insights offer important considerations for the future in relation to holiday club capability to respond to adverse circumstances and adopt new models of working.

Government rules and guidelines implemented to manage the spread of Covid-19, such as national lockdown restrictions, localised rules and restrictions, and the need to universally practise social distancing (25, 26), resulted in holiday club staff needing to make numerous changes to their club delivery methods. Clubs adapted different food delivery methods. Some clubs were able to adapt their on-site food provision in ways that were Covid-compliant. This typically involved replacing buffet-style hot meal provision with lunch bags containing cold food to better manage social distancing and to reduce high-touch areas that could spread Covid-19. The inability to provide a hot meal on-site was disappointing for staff because it reduced opportunities for children, staff, and families to sit and eat together. Commensality, or eating with others, is a social experience (32) that can have positive short and long-term outcomes on children's eating behaviour such as improved healthy eating and reduced fast-food consumption (33). Some clubs were however able to continue to engage children in enrichment opportunities through cooking activities, by reducing the number of children engaged and by allocating children their own set of utensils.

Where on-site food provision became impossible, in line with other charities including the Trussell Trust national foodbank network (7), holiday clubs reverted to delivering food to families in innovative ways such as providing picnics with games and other activities for children, recipe boxes and frozen home cooked meals. These results align with research from the Food Standards Agency (34), which revealed that 9% of the UK population had food donations delivered to their home by a food charity between April and July 2020.

The way in which club staff sought to implement these changes was significant because they intended to promote a sense of agency for families by enabling them to cook healthy and affordable food at home in the long-term by reducing the barriers experienced by many families. When providing food, club staff were also responsive to families' dietary requirements, provided cooking utensils and weighed out ingredients to enable families to cook new recipes that could be replicated in the future.

The innovative changes made by holiday clubs transcended that of providing sustenance and alleviating food poverty. Staff emphasised that holiday clubs are not just about providing food, “*it's about getting involved and then learning those essential life skills*” and “*it's about being proactive and empowering them*” (P7, Programme Manager). Previous research has found that holiday clubs provide a range of health, wellbeing, and developmental opportunities to children and families including alleviating hunger (17), reducing social isolation (21), enhancing social skills (22), and promoting healthy eating (23) and physical activity (21). Holiday clubs enable these opportunities by providing a wealth of services including food, entertainment equipment, sport, physical activity, fun days out, information and education (24). The current study's findings reinforce the role of holiday clubs in supporting families facing hardship, and emphasise the importance of staff maximising their food offer in order to provide many of these broader opportunities (e.g., cooking activities) to children and families, even during adverse circumstances such as the Covid-19 pandemic.

Food insecurity increased significantly during the pandemic resulting in an increased demand for food aid during 2020 (8, 12, 35). In response, most of the clubs adapted both the scale and reach of their provision (theme two). Many staff described how their holiday clubs were able to meet these additional demands for food by adapting their delivery methods (theme one) and bolstering their resources (theme three). However, the provision of food supplies to families reduced in some clubs during the early months of the pandemic, as some clubs had to postpone their on-site delivery and some clubs experienced decreases in resources. This study highlights that holiday clubs were able to provide timely support in response to heightened food insecurity alongside other local and national schemes implemented to alleviate food insecurity, such as the free school meal replacement schemes (36). However, it is clear that these responses were fragmented and *ad-hoc* rather than being part of a strategic response to tackle food insecurity. It is also evident that staff at holiday clubs were focused not only on food provision but also sought to continue to provide their broader offer to feed, stimulate, develop and educate children accessing holiday club provision. Without adequate resources the educational benefits provided by holiday clubs in the most disadvantaged communities are at risk.

Staff also described a range of changes in access to the resources needed to underpin their holiday club food provision. Some clubs were able to access additional resources during the pandemic, including increased funding, volunteers, and food supplies due in part to more partnership working with relevant organisations. However, other clubs reported decreases in financial resources, reductions in staffing due to furloughing, or increases in working demands, and decreased access to club kitchen facilities to prepare the food. Although the UK government announced in April 2020 £750 million of funding to support the charity sector in providing key services during the Covid-19 pandemic, such as food and care provision (37), the resource constraints discussed in this study alongside other food charity research (8, 38) again reinforce the challenges that holiday clubs and other food charities face in accessing and sustaining sufficient resources to underpin their essential provision for families. The UK government is currently negotiating the future funding of holiday clubs by the DfE, with the potential of making funding centralised (24).

Technology provided both opportunities and challenges for the staff seeking to adapt their holiday club provision. Staff in this study highlighted that technology and social media became pivotal following the onset of the Covid-19 pandemic in enabling clubs to provide food and food-related enrichment activities to families when on-site club provision was not possible, and in promoting social connection among families *via* social media. However, utilising technology to maintain connection with families was not without its challenges. The Covid-19 pandemic and its associated measures to contain the virus precipitated a universal reliance on technology in order to remain socially connected (39), and yet, digital exclusion (inequalities in access and use of technology) increased during the pandemic among those from deprived backgrounds with limited access to technology due to device unaffordability, lack of internet access and limited digital skills (40, 41). Similar to this, some staff in this study highlighted that some families had accessibility challenges making it difficult to engage them *via* technology. Staff also highlighted that the use of technology was less appropriate for younger children and that they often struggled to engage children in cooking activities as effectively as when staff delivered sessions on-site. These accessibility challenges placed some families at risk of exclusion in accessing the broader holiday club offer of not only providing food, but also in accessing interactive online activities and in maintaining social connections with other families. Although the DfE sought to alleviate digital inequalities by providing children from disadvantaged communities with access to the internet and digital devices in order to engage with remote learning (42), the digital accessibility challenges highlighted in our study suggests that the UK government should further explore whether its policies to alleviate social inequalities, such as food insecurity and digital exclusion, are sufficiently reaching and supporting the most vulnerable and disadvantaged families. Whilst holiday club staff did use a range of adaptations to maintain their connection with children and their families, the findings indicate that digital exclusion limited what was possible to do for some families. This is significant because these are also the households who will have been most disadvantaged by being excluded from online home-schooling activities. Future research is needed to further understand the impacts of digital exclusion on children and families, and meaningful ways to address these exclusionary experiences.

Partnership working was also shown to be an important facilitator in ensuring that holiday clubs were able to continue to support families. This finding aligns with research from other food charities such as the Trussell Trust (8) who also reported improvements in partnership working to increase funding and public support for foodbanks. Some holiday club staff however noted food waste and food redistribution challenges due to food donations sometimes being towards the end of their shelf life or occasional large volume of snack foods. Our study reveals the important ways in which organisations can work together and highlights opportunities for enhanced partnership working across sectors locally and nationally (43) which could be built upon in future to improve the provision of charitable food (8).

The findings reflect the views of 25 holiday club staff and the study results may not be generalisable to the wider population (44). All the holiday clubs within this study continued to provide food by adapting their provision. It is recognised that adaptation was likely not possible for all holiday clubs across the country, and it is likely that some will have ceased their provision temporarily or permanently, but these clubs did not feature in this study which was focused on continuation of holiday provision. Similarly, it is important to highlight that the results of this study apply to the 25 club staff participants engaged in this study and the nine regions from which they were based; staff experiences may vary in other regions of the UK not represented in this study (i.e., the north east of England, Scotland, and Northern Ireland). It is also a notable limitation that there were no participants from the North East of England, which is one of the most deprived regions in England (45).

An additional limitation is that this study did not explore the experiences of parents and children which could offer insights into whether the adaptations to holiday club food provision have been relevant and accessible from the perspective of families from disadvantaged communities. However, the study does provide important insight into the ways in which holiday clubs adapted quickly and innovatively to meet the challenges that resulted from the pandemic related restrictions that impacted their work at a time when many clubs were attempting to meet the needs of a greater number of families.

This study explored staff perceptions of the adaptations made to holiday club food provision in order to sustain food supplies to families during the first UK national Covid-19 lockdown (March–May 2020) and throughout the 2020 summer holidays. The study also explored the challenges and opportunities faced in making these adaptations. In summary, this study highlights that holiday club staff have an understanding of, and a commitment to, the communities they support which indicates that holiday clubs are ideally placed to support families expediently in times of need. The findings also demonstrate that holiday clubs aim to work with families in ways that recognise that food insecurity is associated with a much broader range of exclusionary experiences than hunger alone, which became amplified by the restrictions imposed in response to the pandemic. Holiday club staff sought not only to provide food to families but also to continue to provide their broader offer to children and their families. Club staff strove to empower families through creating opportunities to engage in a much broader range of activities, such as cooking together, trialling new food and recipes, enjoying outdoor picnics with new equipment, and connecting with others online that may not have otherwise been possible. These activities were particularly significant when viewed against a backdrop of school closures. The study highlights the ability of holiday clubs to adapt but it also indicates that the core mission of holiday clubs to address inequalities for children in disadvantaged communities were threatened as a result of higher levels of food insecurity. While addressing food insecurity in the longer term requires a joined-up strategic response, this study highlights the important role played by holiday clubs in providing an immediate response for families who find themselves in challenging circumstances. Without wider scale reform which addresses the inequalities that drive increasing food insecurity it seems likely that holiday clubs will continue to play pivotal roles in communities for families falling through the food security net. With this in mind, and given the important role that holiday clubs play in their communities as emphasised in this study, the UK government should continue negotiating the future funding of holiday clubs during and beyond the global Covid-19 crisis. Holiday clubs need to be empowered to reliably sustain their club provision to alleviate the rising rates of food insecurity and provide broader enrichment opportunities for some of the most vulnerable children.

## Data Availability Statement

Data are not available on request due to privacy/ethical restrictions (participants did not consent to data sharing). Requests to access the datasets should be directed to Carolynne Mason, C.L.J.Mason@lboro.ac.uk.

## Ethics Statement

The studies involving human participants were reviewed and approved by Loughborough University Ethics Committee (ref: SSEHS-1644). The participants provided their written informed consent to participate in this study.

## Author Contributions

NB led the recruitment and data collection, and contributed to design, analysis, and write up of the manuscript. CM, CH, and EH supervised NB and contributed to the design, analysis, and write up of the manuscript. All authors approved the final manuscript.

## Funding

This research was funded by Loughborough University as part of a fully funded PhD Studentship.

## Conflict of Interest

The authors declare that the research was conducted in the absence of any commercial or financial relationships that could be construed as a potential conflict of interest.

## Publisher's Note

All claims expressed in this article are solely those of the authors and do not necessarily represent those of their affiliated organizations, or those of the publisher, the editors and the reviewers. Any product that may be evaluated in this article, or claim that may be made by its manufacturer, is not guaranteed or endorsed by the publisher.
